# Mozambique Sample Vital Statistics System: Filling the Gaps for Mortality Data

**DOI:** 10.4269/ajtmh.23-0094

**Published:** 2023-04-10

**Authors:** Eduardo Samo Gudo

**Affiliations:** Instituto Nacional de Saúde Moçambique, Maputo, Mozambique

Mortality data, especially concerning deaths by cause, are critical for understanding the burden of disease, planning and monitoring of interventions aimed at reducing morbidity and mortality, as well as monitoring the Sustainable Development Goals for health (goal 3). In sub-Saharan Africa and most limited resource settings where burdens of diseases are high, there is a chronic lack of accurate and reliable data on mortality and causes of death. In these settings, complete diagnostic autopsy is only available in large hospitals and is unaffordable to most people. In addition, most deaths occur at the community level, and for facility deaths, physician medical certification of cause of death using medical information is not systematically completed. Available recent mortality and cause-of-death statistics are mostly based on modeling or periodic national surveys. Although they represent important tools, the uncertainty or long time intervals associated with their estimates challenge their use for precise or timely policy and program planning. Progressive efforts undertaken by low- and middle-income countries (LMICs) to revamp and expand their civil registration and vital statistics (CRVS) systems are commendable and timely. However, the time and resource needs—in terms of infrastructure, technology, equipment, human resources, and demand creation—to reach the entire population with a fully functional CRVS system are enormous and deprioritized in favor of more pressing demands in health care. The recent COVID-19 pandemic and emergence of epidemics across LMICs unveiled the vulnerabilities and challenges associated with the lack of or weak mortality data systems that can support planning, monitoring, and decision-making in these countries.

In this context, building sustainable and resilient mortality surveillance systems that provide high-quality and timely mortality data represents a priority in Africa, as recently expressed in a newly developed continental framework for strengthening mortality surveillance in Africa by the Africa CDC.^1^

The situation in Mozambique is no different from that in other LMICs, where facility-level data on mortality face similar challenges. However, Mozambique launched a major initiative in 2017 to address these challenges, through the development and implementation of a national sample vital statistics system that can routinely produce national and subnational mortality and causes of death data. The initiative, named Countrywide Mortality Surveillance for Action (COMSA), was implemented jointly by the two major national institutions that are responsible for population and health data collection, the National Institute of Statistics (Instituto Nacional de Estatística [INE]) and the National Institute of Health (Instituto Nacional de Saúde [INS]). Technical support was provided by the Johns Hopkins University, and financial support came from the Bill & Melinda Gates Foundation. Before the launch of COMSA in 2017, the country relied on Demographic and Health Surveys, conducted in 1997, 2003, and 2011; a one-time post-census mortality and cause of death survey conducted in 2007; and United Nations modeled statistics for mortality data.

Countrywide Mortality Surveillance for Action is a community-based surveillance system with national and provincial representation, providing a unique opportunity for Mozambique to have accurate and continuous data on mortality, causes of death, and social determinants of death across all age groups. The system targeted 700 randomly selected geographic clusters and trained community workers to report data on pregnancies, pregnancy outcomes, and deaths using mobile phones. More than 60 field data assistants have been trained and are based at the provincial level to supervise community workers and follow up with families of the deceased to collect verbal autopsies. At the central level in Maputo, technical and administrative teams from the INS and the INE monitor the system electronically and conduct frequent visits to the provinces and communities for supervision, monitoring, and capacity strengthening. A robust digital and Web-based system is in place to capture data from the communities and transfer it in real time to the provincial and central levels, where they are reviewed and analyzed. The system allows interrogating the data in real time, analyses to answer relevant questions, data visualizations, and dissemination of the data through a Web portal. Thus, the COMSA system generates all-cause and cause-specific data for use and has established continuous and capacitated teams from communities to the central level for the continuous production of such data. In Mozambique, COMSA is being integrated into the health management information system using the District Health Information Software 2 platform to complement facility level data with community-based data at national and provincial levels. Discussion is also underway to link the system to the national CRVS system to ensure that data collected serves to reinforce the expansion and strengthening of the CRVS system.

## LEVERAGING COMSA FOR DISEASE SURVEILLANCE AND OTHER COMMUNITY ACTIVITIES

As a continuous community surveillance platform, COMSA relies on community surveillance agents who are residents in their cluster or appointed specifically by community leaders. Such bottom-up design gives COMSA an importance that goes beyond mortality data collection. Because of this feature, this platform has the potential to be used in a real-time manner to support other community-based activities, such as disease surveillance, vaccine coverage, other community serological surveys, health promotion, and community sensitization. A serological survey has been successfully implemented using the COMSA platform in Zambezia province, a province with a high number of COMSA clusters due to poor health indicators. Between December 2020 and March 2021, this pilot integrated serological surveillance for vaccine-preventable diseases, malaria, neglected tropical diseases, and other infectious diseases into the routine COMSA data collection. The study aimed to estimate the prevalence and burden of these diseases, to monitor immunity at the population level, and to identify immunity gaps and interventions that cannot be sufficiently characterized through conventional vaccination coverage, disease surveillance, or routine data. This expanded use of COMSA has been shown to increase the programmatic usefulness and sustainability of this platform by expanding the portfolio of funding opportunities.

In Mozambique and other sub-Saharan African countries, the prospective nature of COMSA can allow real-time data collection on several health indicators that otherwise can be obtained only using infrequent cross-sectional countrywide surveys.

## CHALLENGES IMPLEMENTING SUSTAINABLE COMMUNITY-BASED SURVEILLANCE SYSTEMS

Despite its potential to collect prospective data at the community level, complementing facility based data, and filling gaps of cross-sectional surveys, it is important to acknowledge several challenges and limitations, such as the need to 1) ensure that the sampling frame is continuously updated to precisely measure mortality and other relevant health indicators, 2) avoid duplication with other community-based initiatives, 3) ensure timely use of data for policy decisions, and 4) maintain long-term financial sustainability. Although the system can be costly to set up, the running cost can be manageable. Our experience suggests that about two million dollars were required to set up the system, with most of the costs consisting of infrastructure, technology, and training. Once the system was launched, the running costs were cut in half, with two-thirds of the costs for personnel wages and data collection incentives.^2^

## COMSA TRANSITION AND SUSTAINABILITY

As one of the institutions leading COMSA implementation since 2017, INS recognized the importance of having a community-based surveillance system that generates continuous and representative mortality and potentially morbidity data in Mozambique and decided to institutionalize COMSA to support the implementation of disease surveillance and other health-related surveys at the community level. The platform is integrated at the Department of Surveys and Surveillance at INS and is also one of the main sources of data for the National Health Observation, which is a virtual platform aiming to triangulate data from different sources and provide strong evidence on social determinants of health and inequities in health for policy decision-making. Fundraising to ensure its continuation is a priority. As a short-term goal, external funding is being granted for the transition phase, while a sustainability plan is being developed to engage government funds at the medium term. Other opportunities for operational cost-savings while ensuring high-quality and reliable data should continuously be identified in pursuing the long-term sustainability of COMSA. These should include more institutionalization of operations, integration with other interventions, and multi-donor support.
